# Minimal Invasive Circumferential Management of Thoracolumbar Spine Fractures

**DOI:** 10.1155/2015/639542

**Published:** 2015-11-16

**Authors:** S. Pesenti, T. Graillon, N. Mansouri, P. Rakotozanani, B. Blondel, S. Fuentes

**Affiliations:** Spine Unit, Aix-Marseille Université, CHU Timone, 264 rue Saint-Pierre, 13005 Marseille, France

## Abstract

*Introduction*. While thoracolumbar fractures are common lesions, no strong consensus is available at the moment.* Objectives*. The aim of this study was to evaluate the results of a minimal invasive strategy using percutaneous instrumentation and anterior approach in the management of thoracolumbar unstable fractures.* Methods*. 39 patients were included in this retrospective study. Radiologic evaluation was based on vertebral and regional kyphosis, vertebral body height restoration, and fusion rate. Clinical evaluation was based on Visual Analogic Score (VAS). All evaluations were done preoperatively and at 1-year follow-up.* Results*. Both vertebral and regional kyphoses were significantly improved on postoperative evaluation (13° and 7° versus −1° and −9°  *P* < 0.05, resp.) as well as vertebral body height (0.92 versus 1.16, *P* < 0.05). At 1-year follow-up, mean loss of correction was 1°. A solid fusion was visible in all the cases, and mean VAS was significantly reduced form 8/10 preoperatively to 1/10 at the last follow-up.* Conclusion*. Management of thoracolumbar fractures using percutaneous osteosynthesis and minimal invasive anterior approach (telescopic vertebral body prosthesis) is a valuable strategy. Results of this strategy offer satisfactory and stable results in time.

## 1. Introduction

Thoracolumbar fractures consecutive to compression trauma (A fractures in the AO classification) [1] can lead to spine instability and potential neurologic deficits. Furthermore, the collapse of the vertebral body can result, at long follow-up, in a major kyphotic deformity [2]. This kyphotic deformity can therefore be responsible for an overall sagittal anterior malalignment known as a key factor for the development of posttraumatic chronic back pain syndrome [3]. According to these risks, a surgical treatment of these fractures can be advocated in order to restore vertebral body height, correct the kyphotic deformity, and if necessary decompress the neurologic elements [4, 5].

While many treatment options have been described in the literature, there is still a lack of clear consensus regarding the modalities of surgical management for these thoracolumbar unstable fractures. Posterior transpedicular instrumentation is a commonly performed procedure that allows the correction of the kyphotic deformity induced by the fracture. Such spinal fixation can be performed via a traditional open approach or using percutaneous technique. A theoretical advantage of percutaneous techniques is the possibility to avoid complications related to open approach while allowing a same quality of reduction of the posttraumatic kyphotic deformity.

When needed, and especially when there is a high comminution of the vertebral body, an anterior support associated with the posterior instrumentation can be performed in order to obtain an optimal spinal stabilization. Various studies have shown the benefit of the anterior intervertebral stabilization in terms of kyphosis correction or sustainability of this correction at long follow-up [6–8]. With regard to the anterior surgical techniques, different procedures have been described using bone graft, cages, or telescopic vertebral body prosthesis. Such techniques can also be performed using minimal invasive techniques in order to reduce surgical trauma and to improve postoperative course for the patients.

The aim of this study was to report results of our experience in the management of unstable thoracolumbar fractures using a posterior percutaneous transpedicular instrumentation combined with an anterior stabilization using telescopic vertebral body prosthesis.

## 2. Methods

### 2.1. Study Design

In this retrospective study, medical chart of 39 patients (16 women and 23 men) admitted in our institution for thoracolumbar unstable spine fractures was analyzed by an independent observer from surgery. Inclusion criteria were all the patients admitted for unstable thoracolumbar spine fractures without neurologic deficit and treated using percutaneous posterior instrumentation associated with anterior telescopic vertebral body prosthesis, with at least 1 year of follow-up. Indication for anterior approach was based on important vertebral body comminution and/or disc lesion on the preoperative MRI that required anterior column support.

Patients with history of evolutive carcinoma and infectious or inflammatory disease were excluded. Patients with less than 1-year follow-up were also excluded (due to the fact that fusion was assessed on the 1-year follow-up CT-scan).

All the treated fractures were classified using a preoperative CT-scan. A preoperative MRI was also routinely prescribed in order to evaluate intervertebral discs and ligaments, as well as the risk of neurologic impairment due to the fracture. A postoperative CT-scan was also performed in order to confirm the good positioning of the implant and was repeated at one-year follow-up for the evaluation of bone fusion.

### 2.2. Radiographic Measurements

**Figure 1:**
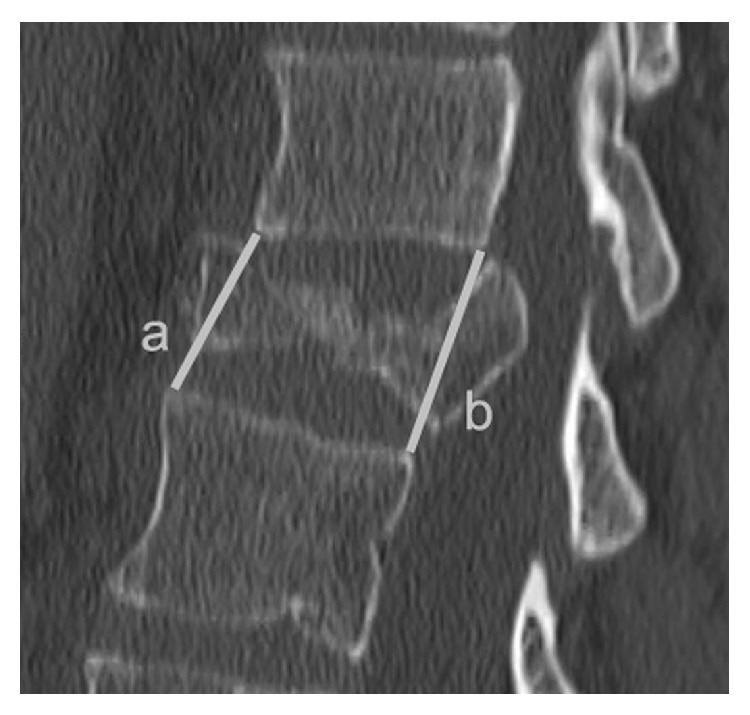
Measurement of the ratio between the anterior and the posterior wall of the collapsed vertebra.

All measurements were performed on pre- and postoperative CT-scan and at last follow-up. Vertebral and regional kyphoses were measured. The regional kyphosis was defined as the angle between the superior endplate of the overlying vertebra and the inferior endplate of the underlying vertebra. The ratio between the anterior and the posterior wall of the collapsed vertebra was also performed (AP ratio) [9] (Figure 1).

The accuracy of pedicle screw placement was also assessed on immediate postoperative CT-scan. A screw was considered as extrapedicular when a cortical breach superior to 2 mm was visible on the postoperative CT-scan [10].

### 2.3. Surgical Technique

On the whole series, surgical strategy was standardized and performed as follows.

**Figure 2:**
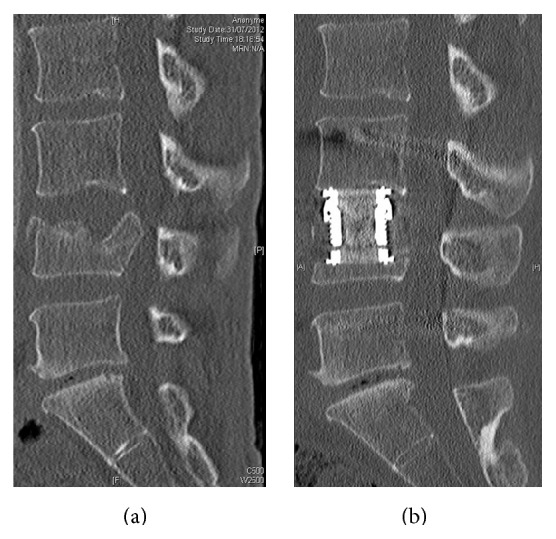
Sagittal pre- (a) and postoperative (b) CT-scan showing a vertebral fracture with a lesion of the superior disc and results after posterior percutaneous osteosynthesis and partial corpectomy.

**Figure 3:**
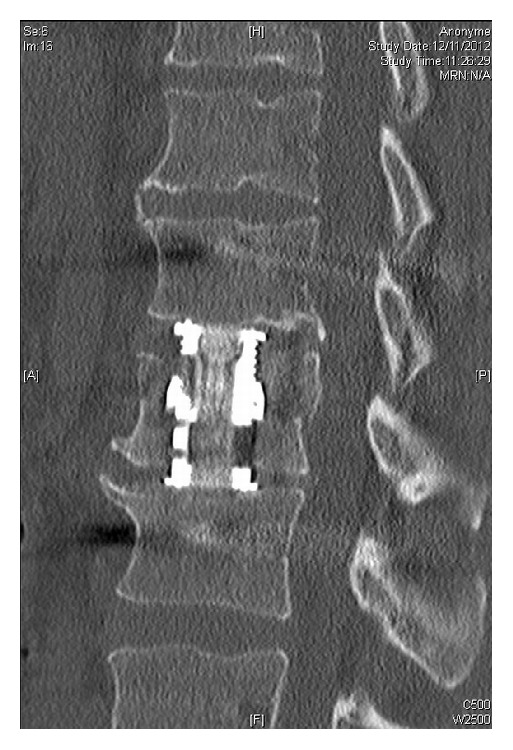
Sagittal postoperative CT-scan showing results after posterior percutaneous osteosynthesis and anterior body graft using an expandable body prosthesis.

- The first step was systematically a posterior percutaneous transpedicular instrumentation using monoaxial screws (Longitude**©**, Medtronic Inc., Memphis, TN), under AP and lateral fluoroscopic control. A short instrumentation was performed in 27 cases (one level above and below the fracture) and a longer instrumentation was performed in 12 cases according to the fracture. Correction of the deformity was achieved using dedicated ancillary and* in situ* contouring techniques.
- The second step was a partial corpectomy (Figure 2) of the collapsed endplate followed by anterior reconstruction using a telescopic prosthetic body (V-lift, Stryker, Kalamazoo, MI), under lateral fluoroscopic control. According to the fracture level, the approach was either a right minithoracotomy (T4–T8), a left minithoracophrenolombotomy (T10-L2), a left minilombotomy (L3-L4), or a retroperitoneal approach (L5) [11]. The prosthetic body was fulfilled with cancellous bone (from the corpectomy) associated with rhBMP-2 (Inductos, Medtronic Inc., Memphis, Tennessee).
- When both adjacent intervertebral discs were affected on the preoperative MRI, the corpectomy was associated with a double discectomy followed by anterior reconstruction using the same telescopic prosthetic body (Figure 3).

The procedure was performed either in one surgical step or in two steps according to patient's comorbidities and associated lesions. In all cases, patients were informed preoperatively about all the surgical and nonsurgical options, the new surgical strategy chosen, and consented accordingly.

### 2.4. Evaluation of Bone Fusion

The assessment of bone fusion was done based on the 1-year follow-up CT-scan. The fusion was considered as acquired when the following criteria were obtained: existence of a bone bridge between over- and underlying vertebras, absence of implant subsidence, absence of hardware failure (anterior or posterior), and absence of osteolytic lesions around the instrumentation (anterior or posterior) [12].

### 2.5. Statistical Analysis

Student's *t*-test was performed to evaluate preoperative to postoperative changes based on radiographic measurements (vertebral and local kyphosis and AP ratio). For each test, the level of significance was set at 5%; that is, *P* values lower than 0.05 were considered to be statistically significant.

## 3. Results

### 3.1. Population

39 patients (23 males and 16 females) with a mean age of 42 years [16–72] were included in this retrospective study. Level distribution of the fractured vertebrae included L1 in 18 cases (46%), T12 in 7 cases (18%), L2 in 6 cases (15%), L4 in 4 cases (10%) and T4, T8, L3, and L5 in 1 case each. According to the AO classification, 25 fractures were classified as A3.3 (64%), 6 as A3.2 (15%), 5 as A3.1 (13%), and 3 as A2 (8%).

Surgical procedure was performed in a single session for 5 patients without comorbidities and associated lesions. In the remaining 34 patients, procedure was achieved in 2 surgical steps according to the fracture, comorbidities, and associated lesions. For patients with a two-step surgical management, anterior approach was done after an average time of 12.3 days [2–47]. A complete corpectomy was performed in 20 cases and a partial corpectomy was performed in the last 19 cases.

Preoperative neurological evaluation did not reveal any deficit in 37 cases. For the 2 last patients, one was classified as Frankel C and one as Frankel D. In these 2 cases, the neurological deficit was not diagnosed immediately due to a concomitant lower limb fracture and anterior approach was done after a 2-day interval.

### 3.2. Kyphosis Reduction

Based on the preoperative CT-scan, mean vertebral kyphosis was measured at 13°  [−8; 36°] and regional kyphosis at 7°  [−37; 26°]. On postoperative examinations (after the posterior and anterior spinal fixation), mean vertebral kyphosis was measured at −1°  [−26; 12°] and regional kyphosis at −8°  [−51; 17°]. For both of these parameters, postoperative reduction was statistically significant (*P* < 0.001) with an average gain of 14° for vertebral kyphosis and 16° for regional kyphosis.

Among the 34 patients managed by a two-step procedure, vertebral and regional kyphoses after the posterior percutaneous instrumentation were measured at 2°  [−4; 10°] and −5°  [−37; 20°] with an average gain of 11° and 13°, respectively. Both vertebral and regional kyphoses were significantly improved after the posterior instrumentation (14° versus 2°  *P* < 0.001 and 9° versus −5°, *P* < 0.001, resp.). Further correction of the kyphotic deformity after the anterior approach was also significant for the vertebral kyphosis and for the regional kyphosis (average gain of 4 and 3°, resp., *P* < 0.05).

At one-year follow-up, mean vertebral kyphosis was −1° and regional kyphosis was −8°, without significant loss of reduction compared to the immediate postoperative evaluation average loss of correction of 1° for vertebral kyphosis (*P* = 0.97) and 1° for regional kyphosis (*P* = 0.85).

### 3.3. Vertebral Body Height Restoration

Comparison between average pre- and postoperative A/P ratio showed a statistically significant (*P* < 0.001) improvement from 0.92 [0.64–1.18] to 1.16 [0.83–1.52].

Among the 34 patients treated during two surgical sessions, A/P ration after the posterior instrumentation was 1.12 on average [0.8–1.35], significantly improved when compared to the preoperative measurement (0.12 versus 1.12, *P* < 0.001). No further significant improvement of the A/P ratio was noticed after the anterior approach (1.12 versus 1.16, *P* = 0.14).

### 3.4. Complications

On the whole series, an unplanned surgical procedure for mechanical complication was never necessary. Based on postoperative CT-scan, the rate of extrapedicular screw (breach superior to 2 mm) was evaluated at 1.8%, without neurologic compromise that required replacement of a screw. Blood loss was inferior to 200 mL in all the cases, only one patient (2.6%) required a blood transfusion during the postoperative course due to associated lesions. At last follow-up, no cases of infection were reported.

For all the patients with a normal preoperative neurological examination, no postoperative deficit was noticed, and a complete recovery was obtained for the 2 patients with preoperative deficits.

### 3.5. Operative Data and Length of Stay

Average operative time was 177 minutes (137 to 263 minutes) when circumferential fusion was performed in the same surgical session. With regard to posterior instrumentation alone, average surgical time was 62 minutes (28 to 99 minutes) and 73 minutes (50 to 105 minutes) for the anterior approach.

Among the 34 patients who underwent two surgical sessions, mean delay between the two procedures was 12 days (2 to 37 days) according to associated lesions.

Average length of stay at the hospital was 15 days (7 to 48 days) dependent on the associated lesions.

### 3.6. Functional Evaluation

On the day of admission, mean VAS was evaluated at 8/10 [3–10]. On the day of discharge of the hospital, mean VAS was at 5/10 and at 1/10 [0–5] at the one-year follow-up evaluation. None of the patients used grade III analgesics at last follow-up.

### 3.7. Fusion Rate

Based on the last follow-up CT-scan, all patients were considered as fused with regard to fusion criteria used for this study and no case of implant failure was noticed.

## 4. Discussion

### 4.1. Surgical Management

Surgical management of thoracolumbar fractures with an important comminution and a kyphotic deformity is nowadays widely accepted. However, there is still a lack of strong consensus of the best strategy in order to achieve best results for these lesions.

Transpedicular posterior instrumentation offers the possibility to achieve a good reduction of the deformity. However, if performed without additional bone graft, posterior instrumentation alone commonly requires an anterior support to reinforce spine stability. It has been advocated by several authors that in case of important bone defect, long-term spinal stability is not guaranteed and a loss of correction up to 10° can occur using posterior instrumentation alone [6–8, 13].

It can therefore be a valuable alternative to perform a circumferential fusion in order to reduce the deformity with stable results [14–16], using a balloon kyphoplasty [17, 18] or an anterior approach [19]. In this series, rationale for an anterior approach instead of balloon kyphoplasty was based on the important bone defect on the initial CT-scan or the presence of a disc disruption on the preoperative MRI.

### 4.2. Percutaneous Osteosynthesis

While conventional open posterior surgery leads to satisfactory results, it can also be a potential source of complications. Among them, intraoperative blood loss and postoperative infection are the most common [20]. Furthermore, it seems that important muscle damage and prolonged retraction can be responsible for local disorders leading to an increased rate of failed back syndrome [21].

A theoretical advantage of percutaneous osteosynthesis is the absence of muscle dissection with decreased blood loss and postoperative infection rate. It has also been demonstrated that satisfactory kyphotic deformity reduction can be obtained using percutaneous approach [22–24]. Results from this study revealed that kyphosis reduction after the posterior instrumentation was satisfactory with a significant restoration of vertebral and regional kyphosis as well as vertebral body height. These results reinforce the previously reported data on the ability of percutaneous procedures that can be associated to in situ contouring for the reduction of kyphotic deformity.

### 4.3. Anterior Corpectomy and Instrumentation

According to our results, regional and vertebral kyphosis was still significantly improved after the anterior approach even if the amplitude of correction was smaller than that after the posterior fixation. This difference can be explained by the distraction effect of the telescopic vertebral body prosthesis.

However, we believe that the clinical impact of this further reduction of the deformity is rather small when compared to the posterior correction. Nevertheless, at one-year follow-up, no significant loss of reduction was visible on CT-scan measurements. These results confirm the interest of anterior approach not to improve the deformity reduction obtained by the posterior instrumentation, but in order to obtain a solid and stable time construct [19].

### 4.4. Fusion Rate

A solid bone fusion was visible in all patients of this series at 1-year follow-up. These results can be related to the absence of important muscle dissection which can be a potential source of pseudoarthrosis [21]. Furthermore, performing a corpectomy with discs resection can increase fusion rate and allow a removal of disc trapped in the vertebral body. Of course, the use of rhBMP-2 is also an important factor in intervertebral fusion rate [25].

### 4.5. Neurologic Decompression

In our series, 2 patients have preoperative incomplete neurologic deficit (Figure 3). Reduction of the kyphotic deformity and distraction maneuvers led, by ligamentotaxis, to a restoration of a normal vertebral canal diameter [26]. Ataka et al. [27] showed that it was possible to restore neurologic function in these patients with incomplete deficits using a posterior only instrumentation without neurological decompression. Using a percutaneous procedure can therefore be a valuable alternative, even for patients with incomplete deficits, as it leads to identical deformity reduction when compared to open procedures.

Furthermore, in this two-step strategy, performing a corpectomy gives a satisfactory access to the vertebral canal. It is therefore possible to obtain via an anterior approach a complete neurologic decompression [28, 29] even more appropriate than a laminectomy due the anterior compression of the neurologic structures.

The presence of an incomplete neurologic deficit is therefore not an absolute contraindication to minimal invasive procedures. It can furthermore be an interesting technique for fragile patients such as polytrauma or elderly.

## 5. Conclusion

Surgical management of thoracolumbar fractures using a percutaneous instrumentation associated with a minimal invasive anterior approach (with a telescopic vertebral prosthesis) leads to a satisfactory and stable reduction of the deformity. While these strategies are commonly used for patients without neurologic deficits, it can also be proposed for patients with incomplete deficits. According to us, this minimal invasive strategy can be a valuable surgical technique in the management of thoracolumbar fractures with a low rate of complications.
